# Assessing the Level of Knowledge About Gastroesophageal Reflux Disease (GERD) in the Riyadh Population: A Cross-Sectional Study in 2025

**DOI:** 10.7759/cureus.97122

**Published:** 2025-11-17

**Authors:** Mohamed Sankari, Waqar Farooqi, Sana A Sankari, Shahid Alnassr, Raghad Zakzouk, Amal AlZaaqi, Saleh Alshehab, Siba A alnassr

**Affiliations:** 1 Faculty of Medicine, Almaarefa University, Riyadh, SAU

**Keywords:** awareness, gastroesophageal reflux, gerd, public knowledge, risk factors

## Abstract

Background: Refluxing stomach contents into the esophagus is the hallmark of gastroesophageal reflux disease (GERD), which results in symptoms and damage to the mucosa. Numerous risk factors are linked to GERD, such as a high body mass index (BMI), *Helicobacter pylori* infection, nonsteroidal anti-inflammatory drug (NSAID) use, dietary practices, and sedentary lifestyles. There has been little research on Saudi Arabian public knowledge of GERD, despite the condition's increasing prevalence. Methods: A cross-sectional study was carried out utilizing an online questionnaire provided via social media channels. The sample size consisted of 318 people living in Riyadh. The questionnaire was derived from validated sources, with questions on demographics, GERD-related knowledge, symptoms, causes, and preventive actions. The data was analyzed using IBM SPSS Statistics, with a significance level of p < 0.05. Results: Out of 318 participants, 68.2% had heard of GERD, but only 25.5% showed good knowledge (≥75% correct responses). Heartburn (79.2%), regurgitation (72.6%), and acid reflux (80.2%) were the most commonly reported symptoms. Key risk factors such as smoking (37.1%) and chronic illnesses (29.6%) were not widely recognized. Females, those with postgraduate education, and persons aged 35-44 showed considerably greater knowledge levels (p < 0.05). Conclusion: Although people in Riyadh are generally aware of GERD, their understanding of its symptoms and risk factors is still lacking. Targeted public health education is essential to enhance early detection and management, particularly for males and younger individuals.

## Introduction

In healthy individuals, occasional reflux of stomach contents into the esophagus is considered physiologically normal. However, when this reflux occurs at least once per week and is accompanied by symptoms such as heartburn or acid regurgitation that damage the esophageal mucosa, it is classified as gastroesophageal reflux disease (GERD) [1]. The gastroesophageal junction plays a central role in the pathophysiology of GERD, with factors such as hiatal hernia contributing to dysfunction of the lower esophageal sphincter (LES) [2]. Prolonged exposure of the esophageal epithelium to gastric acid may further compromise mucosal integrity and lead to complications [3].

GERD presents with symptoms including heartburn, regurgitation, sore throat, and chronic cough [4]. Based on disease severity and duration, GERD is categorized into three types: non-erosive reflux disease (NERD), GERD with erosive esophagitis, and GERD with Barrett’s esophagus, the latter representing a complication of long-standing disease [3]. Beyond symptom burden, GERD can substantially reduce the quality of life and predispose patients to serious complications if untreated [5]. Risk factors include genetic predisposition affecting acid secretion, elevated body mass index (BMI), and *Helicobacter pylori* infection [2]. Additional contributors include the use of nonsteroidal anti-inflammatory drugs (NSAIDs), dietary habits, smoking, family history, sedentary lifestyle, and consumption of highly salted or processed foods [1]. Nonetheless, the precise etiology remains unclear in many cases [6].

In a previous study conducted in Saudi Arabia, it was found that about 75% of Saudis had a good level of awareness regarding GERD [3]. However, there is a notable lack of studies assessing public awareness and knowledge about this disease, despite its increasing prevalence. The global prevalence of GERD has increased in recent decades, resulting in rising healthcare costs and diminished quality of life for affected individuals. If left untreated, GERD can progress to esophagitis, Barrett’s esophagus, and ultimately increase the risk of esophageal adenocarcinoma [7]. In Saudi Arabia, few studies have investigated GERD prevalence. Data from the southwestern region reported a prevalence rate of 32.2%, while a 2016 study estimated a national prevalence of 28.7% [2]. Nocturnal symptoms are also common, affecting up to 72-79% of patients [8].

Prevalence estimates vary according to diagnostic criteria. For example, studies in East Asia report weekly heartburn and/or acid regurgitation symptoms in 2.5-6.7% of the population [5]. Dietary triggers such as spicy food, carbonated beverages, and coffee have been associated with GERD symptoms, although findings regarding coffee remain inconsistent [8]. In the absence of alarm features, GERD diagnosis typically relies on characteristic symptoms, with proton pump inhibitors (PPIs) commonly prescribed as initial empirical therapy [9].

The purpose of this study is to assess public understanding of GERD in Riyadh, Saudi Arabia. Specifically, it aims to evaluate awareness of GERD symptoms, risk factors, and management using a structured questionnaire, identify factors associated with low awareness, and generate evidence to guide targeted educational and preventive strategies. 

## Materials and methods

Study design, setting, and participants

This cross-sectional study was conducted in Riyadh, the capital city of Saudi Arabia. The target population included adult residents of Riyadh, both male and female, aged 18 years and older. Individuals younger than 18 years or residing outside Riyadh were excluded from the study. The participants were recruited through a random sampling technique. The sample size was calculated as 318 participants, based on an estimated GERD prevalence of 29%, a 5% margin of error, and a 95% confidence interval.

Data collection and instrument

Data were collected using a structured, self-administered online questionnaire distributed via Google Forms (Google LLC, Mountain View, California, United States) and shared on various social media platforms (see Appendix). The questionnaire was adapted primarily from Mohammad [3] and consisted of four main sections. The first section covered demographic information, including age, gender, marital status, education level, occupation, and personal or family history of GERD. The second section assessed participants’ knowledge of GERD risk factors, focusing on lifestyle habits, dietary practices, and medication use. The third section explored the frequency and severity of common symptoms such as heartburn and regurgitation, as well as practices related to symptom management. Finally, the fourth section examined lifestyle and dietary behaviors, including smoking, physical activity, lying down after meals, pregnancy, and the consumption of foods commonly associated with GERD, such as chocolate, fried foods, and spicy foods.

Participants achieving a score of ≥70% (≥17 correct answers out of 23) were considered to have good knowledge and practices related to GERD.

Data analysis

Data were cleaned, coded, and analyzed using the IBM SPSS Statistics version 25 (released 2017, IBM Corp., Armonk, NY). Categorical variables were summarized as frequencies and percentages. Statistical significance was assessed using appropriate tests, with a p-value <0.05 considered statistically significant. A Chi-square test was also applied to examine the association between these sociodemographic factors and participants’ knowledge level regarding GERD. 

Ethical considerations

The study protocol was approved by the Institutional Review Board of Almaarefa University (approval no. IRB25-044). Informed consent was obtained from all participants prior to data collection. Participation was voluntary, and respondents had the right to withdraw at any time. Data confidentiality and anonymity were strictly maintained, and all ethical standards were adhered to throughout the study.

## Results

A total of 318 participants were included in this study. Among them, 63.5% were male and 36.5% were female. The majority of participants were aged between 18 and 24 years (45.3%), followed by 25-34 years (26.4%), while 17.6% were aged 45 years or older. Most participants were single (61.6%), had an undergraduate education (51.3%), and were not employed (61.9%). In addition, 33% reported having personally suffered from GERD, and 33% reported a family history of GERD (Table 1).

**Table 1:** Sociodemographic characteristics of the participants This table summarizes the demographic details of the participants, including gender, age group, marital status, education level, employment status, gastroesophageal reflux disease (GERD) status, and family history of GERD.

Parameter		Frequency	Percentage
Gender	Male	202	63.5
	Female	116	36.5
Age (years)	18-24	144	45.3
	25-34	84	26.4
	35-44	34	10.7
	45+	56	17.6
Marital status	Single	196	61.6
	Married	122	38.4
Education	Secondary school	99	31.1
	Undergraduate	163	51.3
	Postgraduate	56	17.6
Work status	Working	121	38.1
	Not working	197	61.9
Have you suffered from GERD?	Yes	105	33.0
	No	213	67.0
Family history of GERD	Yes	105	33.0
	No	213	67.0

Knowledge and awareness of GERD

**Figure 1:**
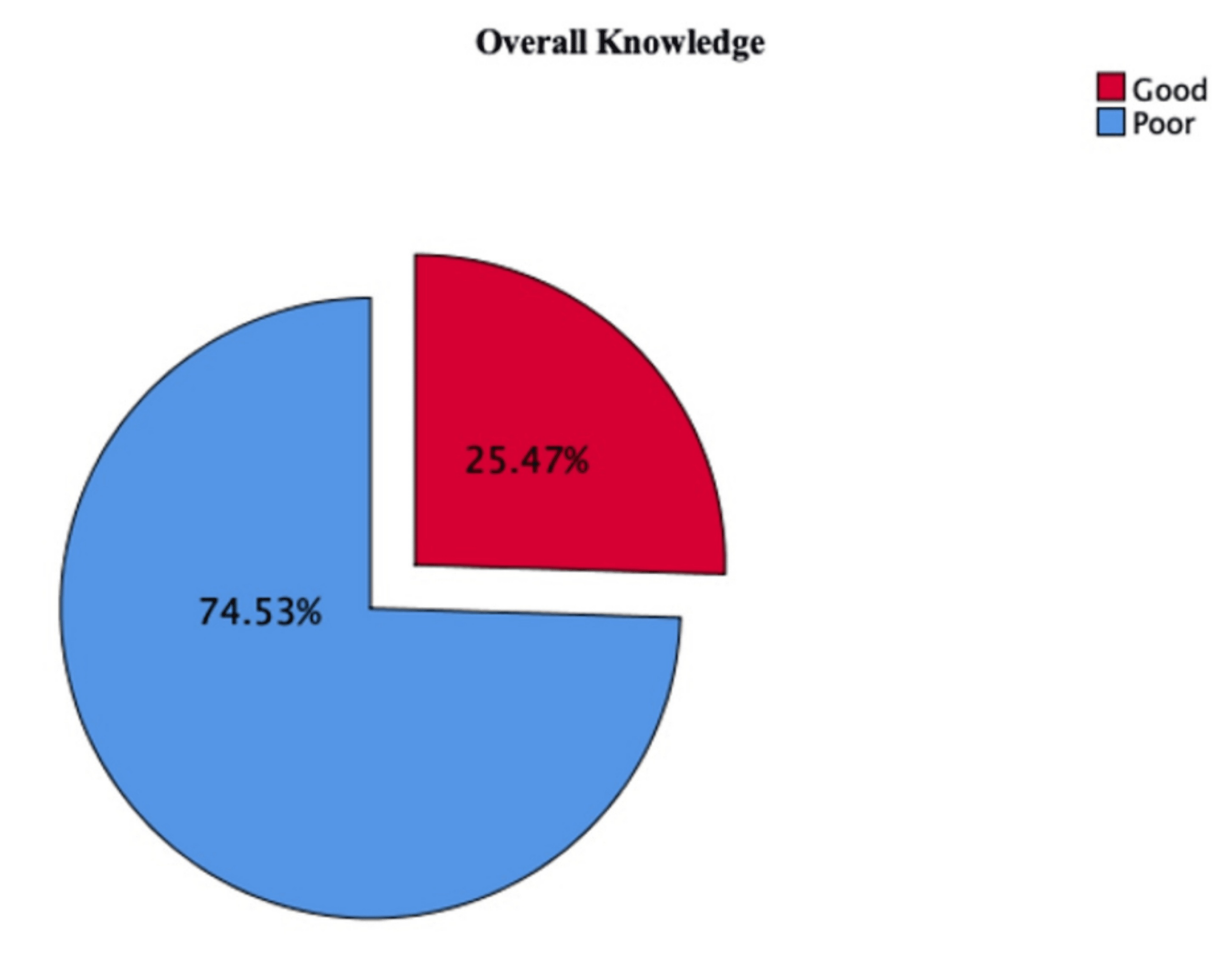
Overall proportion of participants with good and poor knowledge regarding GERD This pie chart shows the percentage of participants catagorized as having good or poor knowledge about gastroesophageal reflux disease (GERD) based on a 70% cutoff score.

The mean knowledge score was 1.25 ± 0.44. Overall, 68.2% had heard of the term “GERD,” while 42.1% were aware that recurrent sore throat might be a symptom. A majority (62.6%) identified carbonated drinks and caffeine as risk factors, while 73.0% recognized that lying down immediately after meals could increase the risk. However, awareness was lower regarding snacking (32.4%), pregnancy (46.5%), and old age (47.8%) as risk factors (Table 2). Figure 1 presents the overall knowledge distribution, where the majority of participants (75.8%) had poor knowledge, and only 24.2% demonstrated good knowledge.

**Table 2:** Knowledge, attitudes, and practices regarding GERD This table displays the frequency and percentage of participants' responses regarding awareness of various risk factors for gastroesophageal reflux disease (GERD).

Parameter		Options	F(%)
Knowledge	Have you ever heard about the term "gastroesophageal reflux disease" or "GERD"?	Yes	217 (68.2)
		No	74 (23.3)
		I don’t know	27 (8.5)
	Are you aware that repeated episodes of sore throat may indicate GERD?	Yes	134 (42.1)
		No	80 (25.2)
		I don’t know	104 (32.7)
	Do you think increased intake of carbonated drinks and caffeine can cause GERD?	Yes	199 (62.6)
		No	41 (12.9)
		I don’t know	78 (24.5)
	Do you think that GERD can be due to frequent snacking?	Yes	103 (32.4)
		No	121 (38.1)
		I don’t know	94 (29.6)
	Does old age increase the risk of GERD?	Yes	152 (47.8)
		No	64 (20.1)
		I don’t know	102 (32.1)
	Do you agree that lying down immediately after having a meal may increase the risk of GERD?	Yes	232 (73.0)
		No	28 (8.8)
		I don’t know	58 (18.2)
	Pregnant ladies are at a higher risk of developing GERD.	Yes	148 (46.5)
		No	30 (9.4)
		I don’t know	140 (44.0)
Attitude	Do you think family history can be a risk factor for GERD?	Yes	124 (39.0)
		No	97 (30.5)
		I don’t know	97 (30.5)
	Do you think intake of a healthy diet in routine can help prevent GERD from developing?	Yes	248 (78.0)
		No	27 (8.5)
		I don’t know	43 (13.5)
	Do you think GERD can be avoided by maintaining a healthy BMI?	Yes	226 (71.1)
		No	31 (9.7)
		I don’t know	61 (19.2)
	Do you think regular exercise can help in the prevention of GERD?	Yes	244 (76.7)
		No	22 (6.9)
		I don’t know	52 (16.4)
Practice	Do you have a close family member affected by GERD?	Yes	120 (37.7)
		No	142 (44.7)
		I don’t know	56 (17.6)

Awareness of GERD signs and symptoms

Heartburn (79.2%), acid reflux (80.2%), and regurgitation (72.6%) were the most recognized symptoms among the participants. Conversely, awareness was significantly lower regarding symptoms such as dental problems (31.1%), chest pain (54.7%), and difficulty or pain with swallowing (49.4%). Notably, only 23.6% associated itching with GERD, indicating a lower level of awareness for atypical presentations (Table 3). 

**Table 3:** Awareness of signs and symptoms associated with GERD This table presents the frequency and percentage of participants’ responses regarding awareness or various gastroesophageal reflux disease (GERD) risk factors.

Parameter		Options	F(%)
Signs and symptoms	Regurgitation	Yes	231 (72.6)
		No	56 (17.6)
		I don’t know	31 (9.7)
	Itching	Yes	75 (23.6)
		No	162 (50.9)
		I don’t know	81 (25.5)
	Heartburn	Yes	252 (79.2)
		No	42 (13.2)
		I don’t know	24 (7.5)
	Acid reflux	Yes	255 (80.2)
		No	35 (11.0)
		I don’t know	28 (8.8)
	Chest pain	Yes	174 (54.7)
		No	95 (29.9)
		I don’t know	49 (15.4)
	Difficulty and pain with swallowing	Yes	157 (49.4)
		No	100 (31.4)
		I don’t know	61 (19.2)
	Dental problems	Yes	99 (31.1)
		No	136 (42.8)
		I don’t know	83 (26.1)

Awareness of GERD causes 

Regarding risk factors, 60.1% correctly identified *Helicobacter pylori* infection as a possible cause of GERD, while only 37.1% acknowledged smoking as a contributing factor. Awareness was modest for muscular abnormalities of the digestive tract (50.3%), and notably low for chronic conditions such as diabetes and hypertension (Table 4).

**Table 4:** Awareness of causes associated with GERD This table illustrates participants' awareness regarding causes of gastroesophageal reflux disease (GERD), including behavioural and medical conditions.

Parameter		Options	F(%)
Causes	Smoking	Yes	118 (37.1)
		No	98 (30.8)
		I don’t know	102 (32.1)
	Infections of *Helicobacter pylori*	Yes	191 (60.1)
		No	64 (20.1)
		I don’t know	63 (19.8)
	Chronic diseases like diabetes or hypertension	Yes	94 (29.6)
		No	124 (39.0)
		I don’t know	100 (31.4)
	Muscular abnormalities in the digestive tract	Yes	160 (50.3)
		No	66 (20.8)
		I don’t know	92 (28.9)

Attitude and preventive practices

Most participants believed that maintaining a healthy BMI (71.1%) and engaging in regular exercise (76.7%) could help prevent GERD. In addition, 78.0% agreed that a healthy diet plays a role in prevention. However, only 39.0% considered family history a contributing risk factor (Table 2). 

Association between sociodemographic factors and knowledge level

**Figure 2:**
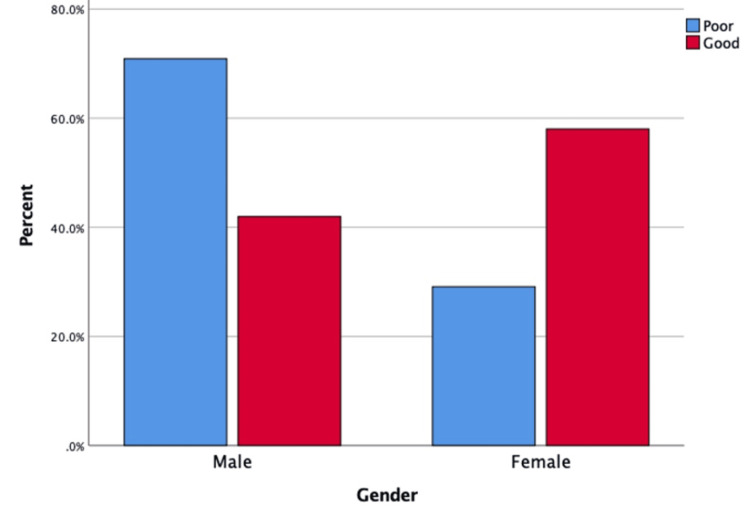
Distribution of knowledge level regarding GERD by gender (N= 318) This bar chart illustrates the proportion of participants with good and poor knowledge about gastroesophageal reflux disease (GERD), stratified by gender.

**Figure 3:**
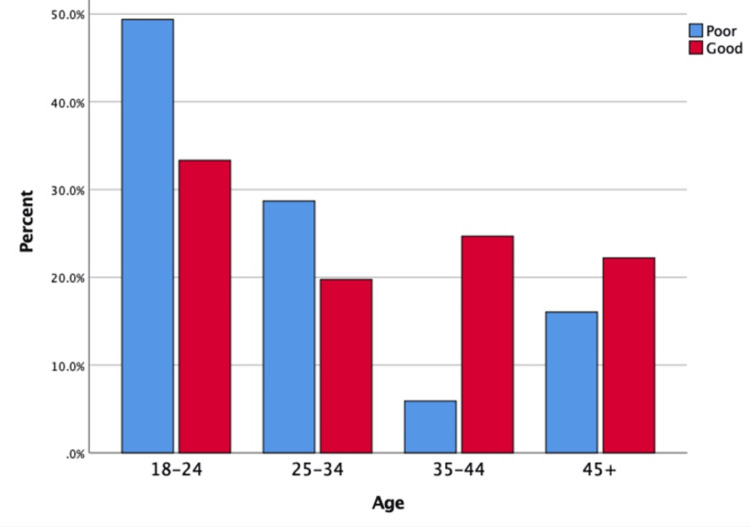
Distribution of knowledge level regarding GERD by age group (years) (N = 318) This bar chart displays the percentage of participants with good and poor knowledge of gastroesophageal reflux disease (GERD) across the age groups (18–24, 25–34, 35–44, +45 years).

**Figure 4:**
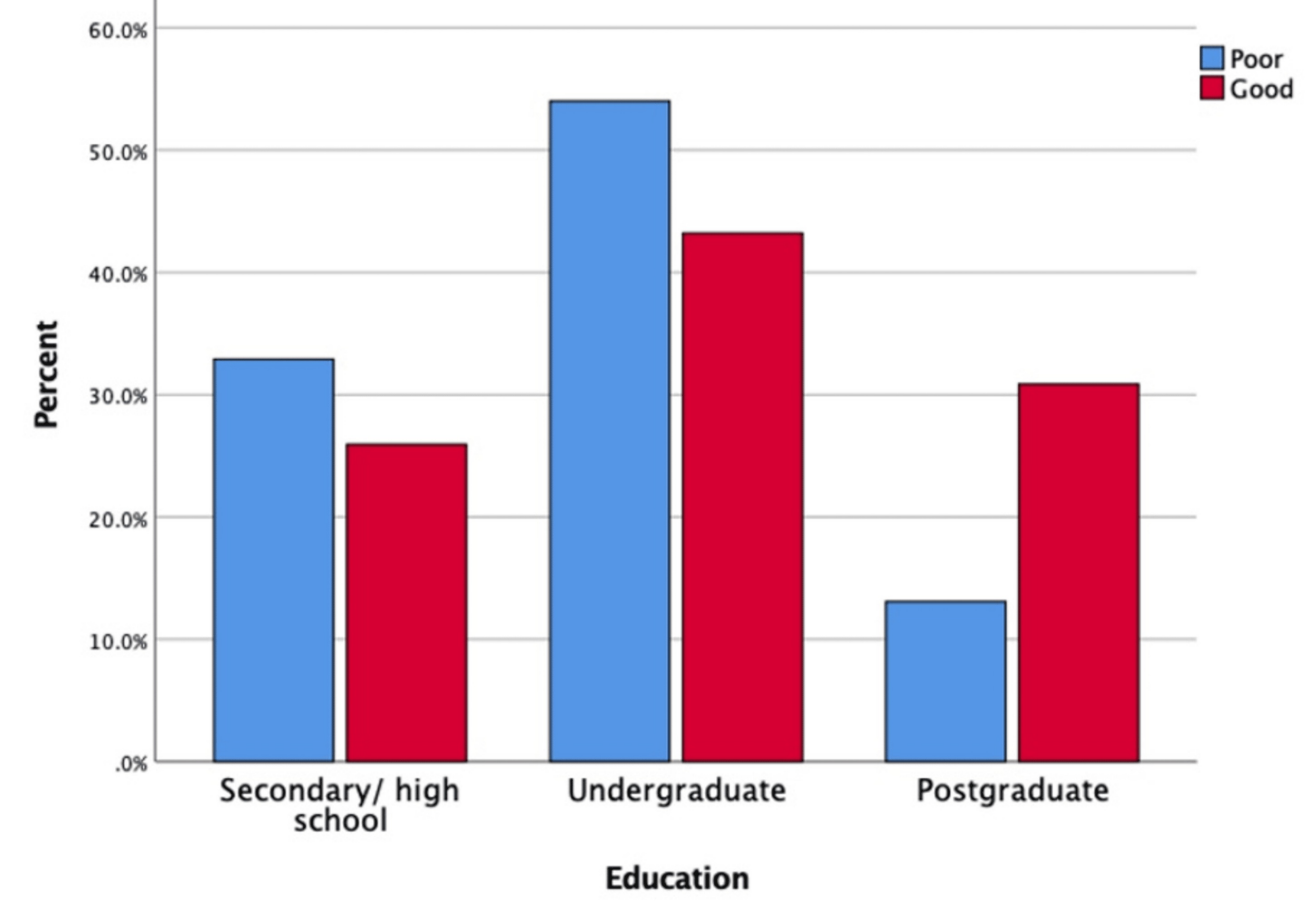
Distribution of knowledge level regarding gastroesophageal reflux disease (GERD) by education level (N= 318) This bar chart shows the knowledge levels (good vs. poor) among participants categorized by education level: secondary, undergraduate, and postgraduate.

A statistically significant association was observed between knowledge level and several sociodemographic factors. Female participants demonstrated significantly higher levels of good knowledge compared to males (40.5% vs. 16.8%, p = 0.001), as illustrated in Figure 2. Participants aged 35-44 years had the highest percentage of good knowledge (58.8%, p = 0.001), as shown in Figure 3. Postgraduate education was significantly associated with higher knowledge levels (44.6%, p = 0.001), as depicted in Figure 4. Marital status also showed a significant relationship, with married individuals showing better knowledge (32.8%, p = 0.018). There was no significant association between work status and knowledge (p = 0.399). However, family history of GERD was significantly associated with higher knowledge levels (p = 0.011) (Table 5).

**Table 5:** Association between sociodemographic factors and knowledge level regarding GERD This table presents the association between sociodemographic factors and participants’ knowledge levels regarding gastroesophageal reflux disease (GERD). The knowledge score was categorized based on a 70% cutoff, where participants scoring ≥17 out of 23 were classified as having good knowledge, and those scoring below were classified as having poor knowledge. Statistical test used: Chi-square test of independence

		Knowledge			
Parameter		Good	Poor	P-value	Chi-square
Gender	Male	34 (16.8%)	168 (83.2%)	0.001	21.776
	Female	47 (40.5%)	69 (59.5%)		
Age	18-24	27 (18.8%)	117 (81.3%)	0.001	26.488
	25-34	16 (19.0%)	68 (81.0%)		
	35-44	20 (58.8%)	14 (41.2%)		
	45+	18 (32.1%)	38 (67.9%)		
Marital status	Single	41 (20.9%)	155 (79.1%)	0.018	5.580
	Married	40 (32.8%)	82 (67.2%)		
Education	Secondary School	21 (21.2%)	78 (78.8%)	0.001	13.161
	Undergradute	35 (21.5%)	128 (78.5%)		
	Postgraduate	25 (44.6%)	31 (55.4%)		
Work status	Working	34 (28.1%)	87 (71.9%)	0.399	0.710
	Not working	47 (23.9%)	150 (76.1%)		
Have you suffered from GERD?	Yes	33 (31.4%)	72 (68.6%)	0.087	2.930
	No	48 (22.5%)	165 (77.5%)		
Family history of GERD	Yes	36 (34.3%)	69 (65.7%)	6.415	0.011
	No	45 (21.1%)	168 (78.9%)		

## Discussion

This study demonstrated a generally high level of awareness regarding major risk factors for GERD. The majority of participants correctly identified the consumption of carbonated beverages and caffeine, lying down immediately after meals, and the benefits of maintaining a healthy diet and BMI through regular physical activity as key factors influencing GERD risk. Nearly half also recognized pregnancy and older age as contributors to GERD development. This finding aligns with research from Al-Taif, Saudi Arabia, where 64.9% of the respondents acknowledged pregnancy as a risk factor for GERD [6].

However, awareness of other lifestyle-related factors, particularly frequent snacking, was considerably lower in the present study. By contrast, Mohammad SM et al. [3] reported in 2021 that 78% of participants recognized frequent snacking as a risk factor, while Alrashed AA et al. [4] found that 56.5% of Saudi university students who frequently snacked experienced GERD symptoms. These variations may reflect evolving dietary habits among younger populations and the normalization of unhealthy eating behaviors over time.

In terms of symptom recognition, the participants exhibited limited awareness of atypical GERD symptoms such as itching and dental problems. This observation is consistent with the findings of Mohammad SM et al. [3], who reported that only 3.1% of respondents identified itching as a GERD-related symptom. The low recognition of dental manifestations may be explained by their relatively low prevalence. For instance, Otayf B et al. [10] reported a 2.3% prevalence of dental problems among students at Jazan University. These results highlight the need for public health initiatives to improve understanding of less common GERD symptoms while reinforcing knowledge of typical manifestations such as regurgitation, heartburn, acid reflux, and chest pain, which were widely recognized by participants.

Regarding the etiologies of GERD, over half of the respondents identified *Helicobacter pylori *infection as a contributing factor, indicating a possible improvement in public awareness compared to earlier findings by Mohammad SM et al. [3], where only 31.2% recognized this association. However, awareness of other causes, including chronic diseases and smoking, remained limited. This contrasts with findings from Riyadh by Mahmoud MH et al. [7], where 60.1% identified smoking as a risk factor. Supporting its significance, Halawani H and Banoon S [2] reported that 57.9% of smokers in the Makkah region had been diagnosed with GERD, emphasizing smoking as a major modifiable risk factor. These findings underscore the importance of incorporating smoking cessation strategies into broader public health campaigns aimed at GERD prevention.

Sociodemographic factors, including age, gender, and education level, were significant predictors of GERD knowledge. The highest awareness levels were observed among individuals aged 35-44 years and those with postgraduate education, whereas males and younger adults aged 18-24 demonstrated the lowest awareness. Interestingly, participants with a personal or family history of GERD did not exhibit significantly greater knowledge than those without such experiences. This suggests that healthcare providers may be missing key opportunities to educate patients and their families during clinical encounters. Strengthening patient education during medical consultations may therefore play a pivotal role in improving public understanding of GERD and its risk factors.

Limitations

This study has several limitations that should be considered when interpreting the findings. First, the cross-sectional design limits the ability to establish causal relationships between sociodemographic factors and participants’ knowledge of GERD. Second, because the study was conducted exclusively in Riyadh, the results may not be generalizable to populations in other regions with different cultural norms, dietary practices, or access to healthcare services. Finally, the absence of objective diagnostic measures, such as endoscopy or pH monitoring, restricts the ability to correlate participants’ reported awareness with clinically confirmed cases of GERD.

## Conclusions

This study assessed public knowledge of GERD among residents of Riyadh, Saudi Arabia. Although 68.2% of respondents reported having heard of GERD, only 25.47% demonstrated adequate knowledge of the condition. These findings reveal a considerable gap in public understanding, particularly among younger individuals, males, and those with lower educational attainment. Targeted health education campaigns are therefore needed to improve awareness of GERD, its risk factors, and potential complications. Enhanced public knowledge can support earlier diagnosis, reduce disease burden, and improve quality of life. Notably, the lack of increased awareness among individuals with a personal or family history of GERD highlights missed opportunities for patient education during clinical encounters.

## Appendices

Study questionnaire

You are invited to participate in a research study aimed at studying the level of knowledge about gastroesophageal reflux disease (GERD) in the Riyadh population.

Your participation in the research study is voluntary; you can choose not to participate. All private information in this questionnaire will be used for research purposes only. The time taken to fill out the questionnaire is approximately 5 minutes. Wishing you health and wellness.

First Section: Demographics

1) Age (years) 

✓ 18−24

✓ 25− 34

✓ 35− 44

✓ 45+

2) Gender

✓Male

✓Female

3) Education 

✓Secondary/ high school

✓Undergraduate

✓Postgraduate

4) Work status 

 ✓Not working

 ✓Working

5) Marital status

 ✓Single 

 ✓Married

6) Do you suffer from GERD?

✓Yes

✓No

7) Do you have a family history of GERD

✓Yes

✓No

Second Section: Awareness of the Risk Factors Related to GERD

1) Have you ever heard about the term "gastroesophageal reflux disease" or "GERD"? 

✓ Yes

✓ No

✓ I don’t know

2) Do you have a close family member affected by GERD?

✓ Yes

✓ No

✓ I don’t know

3) Do you think family history can be a risk factor for GERD?

✓ Yes

✓ No

✓ I don’t know

4) Are you aware that repeated episodes of sore throat may indicate GERD?

✓ Yes

✓ No

✓ I don’t know

5) Do you think increased intake of carbonated drinks and caffeine can cause GERD? 

✓ Yes

✓ No

✓ I don’t know

 6) Do you think that GERD can be due to frequent snacking?

✓ Yes

✓ No

✓ I don’t know

7) Does Old age increase the risk of GERD?

✓ Yes

✓ No

✓ I don’t know

8) Do you think the intake of a healthy diet in routine can help prevent  GERD development? 

✓ Yes

✓ No

✓ I don’t know

9) Do you agree that lying down immediately after having a meal may increase the risk of GERD? 

✓ Agree

✓ Disagree

✓ I don’t know

10) Pregnant ladies are at a higher risk of developing GERD.

✓ Agree

✓ Disagree

✓ I don’t know

11) Do you think GERD can be avoided by maintaining a healthy BMI?

✓ Agree

✓ Disagree

✓ I don’t know

12) Do you think regular exercise can help in the prevention of GERD? 

✓ Agree

✓ Disagree

✓ I don’t know

Third Section: Which of These Symptoms Do You Think Are Related to GERD?

1) Regurgitation 

✓ Yes

✓ No

✓ I don’t know

 2) Itching 

✓ Yes

✓ No

✓ I don’t know

3) Heartburn 

✓ Yes

✓ No

✓ I don’t know

4) Acid reflux

✓ Yes

✓ No

✓ I don’t know

5) Difficulty and pain with swallowing  

✓ Yes

✓ No

✓ I don’t know

6) Chest pain 

✓ Yes

✓ No

✓ I don’t know

7) Dental problems

✓ Yes

✓ No

✓ I don’t know

Fourth Section: Which of the Conditions Do You Think Can Cause GERD?

1) Chronic diseases

✓ Yes

✓ No

✓ I don’t know

2) Smoking

✓ Yes

✓ No

✓ I don’t know

3) Infections of *Helicobacter pylori*

✓ Yes

✓ No

✓ I don’t know

4) Muscular abnormalities in the digestive tract

✓ Yes

✓ No

✓ I don’t know 

## References

[1] Alkhathami AM, Alzahrani AA, Alzhrani MA, Alsuwat OB, Mahfouz ME (2017). Risk factors for gastroesophageal reflux disease in Saudi Arabia. Gastroenterology Res.

[2] Halawani H, Banoon S (2020). Prevalence and determinants of gastroesophageal reflux disease and the risk factors among adult patients attending Al-Iskan Primary Health Care Center in Makkah, 2020. Cureus.

[3] Mohammad SM, Mrair AA, Alqaraishi AM (2021). General public awareness toward gastroesophageal reflux disease in Saudi Arabia. Int J Med Dev Ctries.

[4] Alrashed AA, Aljammaz KI, Pathan A, Mandili AA, Almatrafi SA, Almotire MH, Bahkali SM (2019). Prevalence and risk factors of gastroesophageal reflux disease among Shaqra University students, Saudi Arabia. J Family Med Prim Care.

[5] Alsulobi AM, El-Fetoh NM, Alenezi SG (2017). Gastroesophageal reflux disease among population of Arar City, Northern Saudi Arabia. Electron Physician.

[6] Taha N, Sahaki M, Maimsh O (2018). Assessment of the knowledge of gastroesophageal reflux disease among the Saudi population of Altaif City. Egypt J Hosp Med.

[7] Mahmoud MH, Alshalawi AAM, Alasmi STS (2020). Awareness of Saudi population about symptoms and predisposing factors of gastroesophageal reflux. Int J Med Dev Ctries.

[8] Alsaleem MA, Awadalla NJ, Shehata SF (2021). Prevalence and factors associated with gastroesophageal reflux disease among primary health care attendants at Abha city, southwestern Saudi Arabia. Saudi Pharm J.

[9] Baklola M, Terra M, Badr A (2023). Prevalence of gastro-oesophageal reflux disease, and its associated risk factors among medical students: a nation-based cross-sectional study. BMC Gastroenterol.

[10] Otayf B, Dallak F, Alomaish A, Qadri A, Moafa R, Gosadi I, Alhazmi AH (2022). Prevalence and risk factors of gastroesophageal reflux among Jazan University students, Saudi Arabia: a cross-sectional study. Cureus.

